# Distribution, maturity and population structure of *Meganyctiphanes norvegica* and *Thysanoessa inermis* around Iceland in spring

**DOI:** 10.1371/journal.pone.0187360

**Published:** 2017-11-07

**Authors:** Teresa Silva, Astthor Gislason, Olafur S. Astthorsson, Gudrún Marteinsdóttir

**Affiliations:** 1 Pelagic Division, Marine and Freshwater Research Institute, Skulagata 4, Reykjavik, Iceland; 2 Institute of Biology, University of Iceland, Sturlugata 7, Reykjavik, Iceland; 3 Environment Division, Marine and Freshwater Research Institute, Skulagata 4, Reykjavik, Iceland; Evergreen State College, UNITED STATES

## Abstract

This study aims to explain the distribution, maturity and population structure of *Meganyctiphanes norvegica* and *Thysanoessa inermis* in springtime in relation to main hydrographic regions around Iceland: Atlantic in the southwest, Atlantic-Arctic mixture in the north and Arctic in the east. Krill were collected 14–29 May 2013 using a macrozooplankton trawl. Biomass of both species combined was significantly higher in the southwest than in north and east. *M*. *norvegica* clearly dominated in Atlantic waters, whereas *T*. *inermis* was more evenly distributed around the island, while the highest values were also observed in the southwest for this species. Simple linear regressions showed that the abundance of *M*. *norvegica* was positively related to temperature, salinity and phytoplankton concentration, while the abundance of *T*. *inermis* was negatively related to bathymetry. Multiple linear regression analyses did not add to this information of a positive relationship between abundance and temperature for *M*. *norvegica*, while *T*. *inermis* was shown to be negatively related to both temperature and bathymetry. During the latter half of May, the main spawning of both species was confined to the regions off the southwest coast. Sex ratio (males/females) of *M*. *norvegica* was higher in the southwest than in the north and east, whereas *T*. *inermis* showed a similar sex ratio all around the island. In all regions, *M*. *norvegica* appears to have a lifespan of 2 years while *T*. *inermis* of 1 year in the southwest and possibly 2 years in north and east.

## Introduction

As in most other areas of the North Atlantic, krill play an important role in the Icelandic marine food web, as conveyors of energy from lower to higher trophic levels including several commercially exploited fish species, seabirds and marine mammals [1–7]. Astthorsson et al. [8] demonstrated that krill appeared to be the third most important taxonomic group within the Icelandic exclusive economic zone (EEZ) in terms of biomass, with an estimated annual wet weight of ~5 million tons. Despite the importance of krill in the Icelandic marine ecosystem, there is limited information on the large-scale distribution of krill in Icelandic waters. Mostly because sampling krill in this environment at appropriate spatio-temporal scales is logistically and financially challenging.

In the North Atlantic, *Meganyctiphanes norvegica* and *Thysanoessa inermis* are important krill species in terms of abundance and biomass [1,9–15]. Both species usually inhabit the upper 400 m of the water column [12,13,16,17]. A third species, *T*. *longicaudata* is also common in the North Atlantic (e.g. Saunders et al. [13]). However, due to its much smaller size than the other two, it was not sampled effectively by the present sampling. *M*. *norvegica* is distributed from the Mediterranean Sea northwards to the subarctic waters in the Norwegian and Greenland Seas [16], whereas *T*. *inermis* is mainly found in coastal and shelf break waters of the northern part of the North Atlantic and the Pacific [9, 16].The first systematic study of krill in Icelandic waters is that of Paulsen [18], who recorded occurrence and distribution. Stephensen [19] later gave an overview of what was then known of the biology and distribution of krill species around Iceland. Later, Einarsson [9] reviewed the distribution and ecology of krill around the island, however, mostly confined to coastal waters. The other earlier studies on krill were restricted to specific geographic locations in the north [10,11] and southwest of Iceland [1].

The earlier studies from Icelandic waters indicate that the distribution of *M*. *norvegica* is mainly restricted to the shelf break waters off the southwest coast, with the main spawning regions located over the slope areas in the southwest [9]. *T*. *inermis*, on the other hand, is reported as common on the coastal banks all around the island with the main spawning usually occurring in the coastal waters of the northern and eastern coasts [9]. Einarsson’s [9] study is widely recognized as a benchmark study for euphausiid ecology across the North Atlantic. However, as stated above most of his samples were collected from coastal waters which has restricted our understanding of the ecology of krill species in the study region. This study seeks to resolve this issue by extending observations into the offshore environment around Iceland.

**Fig 1:**
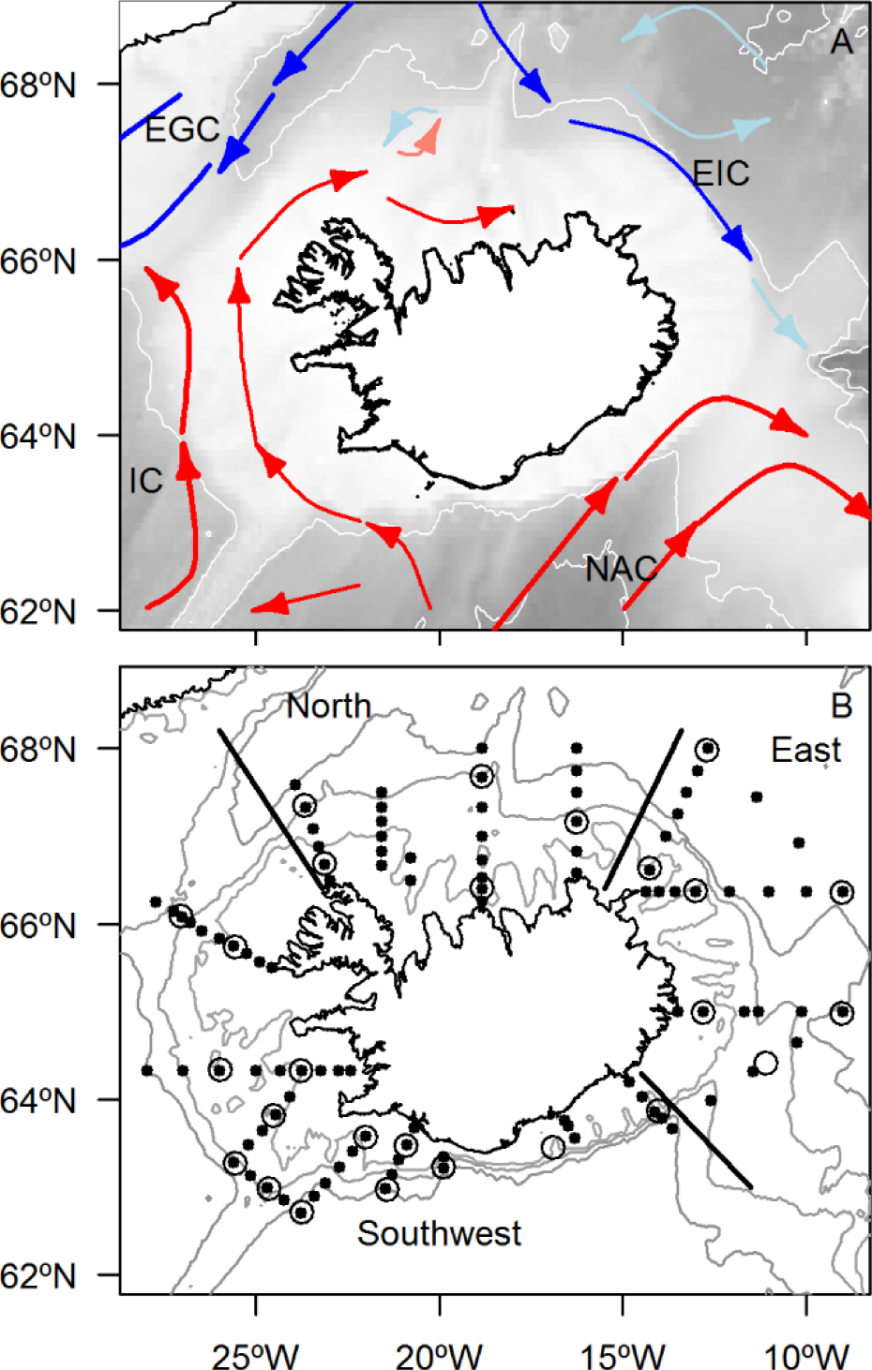
Study area. (A) Map displaying the main ocean currents around Iceland. The currents are adapted from Valdimarsson and Malmberg [33]. NAC: North Atlantic Current; IC: Irminger Current; EGC: East Greenland Current and EIC: East Iceland Current. (B) The locations of monitoring stations during the Icelandic spring survey 14–29 May 2013. The black points indicate the CTD stations and open circles show locations where the macrozooplankton trawl was deployed. Grey lines show isobaths of 200, 500 and 1000 m.

Iceland is located on a system of submarine ridges which influences the flow of ocean currents and the distribution of water masses around the island [20–26]. The main ocean currents are the East Greenland Current and the East Icelandic Current that transport cold water to the north and east of Iceland and the North Atlantic Current and the Irminger Current that carry warm Atlantic water to the south and west of Iceland (Fig 1A). Based on hydrographic characteristics Icelandic waters may be divided into three distinct regions: southwest where Atlantic water prevails, north where a mixture of Atlantic-Arctic water dominates and east where the influence of Arctic water is most pronounced [27–29]. Although the system is highly dynamic and inter-connected by ocean currents, previous studies have shown that the different hydrographic regions at least partly structure both phytoplankton [30] and zooplankton [27–29, 31, 32] communities around the island. Given the role of krill in the marine food web, it is clearly of interest to study the large-scale distribution and population dynamics of krill in the region. This study aims to describe the distribution, maturity and population structure of *M*. *norvegica* and *T*. *inermis* around Iceland. The results will be examined in the context of the division into main hydrographic domains described above (Fig 1).

## Materials and methods

### Ethics statement

All necessary permits for this study were issued by the Icelandic government to the Marine and Freshwater Research Institute, Iceland. We confirm that the field studies did not involve endangered or threatened species.

### Sampling

Sampling took place as part of the annual spring survey of the Marine and Freshwater Research Institute during 14–29 May 2013 on the R.V. *Bjarni Sæmundsson*. Samples of krill were obtained using a large fine-meshed midwater trawl, ‘macrozooplankton trawl’ with a 27-m^2^ mouth opening made of dynice line. The mesh size of the trawl net is identical from the trawl opening to the codend (knotless nylon net with 4 mm meshes, 6 mm stretched). The trawl is equipped with relatively short lastridge lines that causes the trawl net to undulate when towed through the water, preventing animals from being enmeshed in it. The trawl has short 800 mm mesh size wings with floats at the upper wing linings and chains as weight at the lower linings. Floats are also fastened to the headline and chains to the bosom (i.e. the centre portion). The trawl is spread by trawl doors that are fastened to the wing ends by 30 m long dynice towing ropes.

The samples were taken at 13 transects running approximately perpendicular to the coast (Fig 1B). Usually, two trawl stations were occupied on each transect, one over the shelf and the other offshore (Fig 1B). A total of 26 trawl stations were undertaken during the study, with sampling conducted during both day (19 stations) and night (7 stations). The trawl was towed obliquely at a speed of ~1.5–2 knots from 200 m to the surface or ~10 m from the bottom where the bottom depth was less than 200 m. A Scanmar acoustic trawl sensor (Marport’s Compact Trawl Explorer) was placed on the headline of the trawl to monitor depth and the vertical opening of the trawl during the tow. The volume filtered by the trawl was estimated by multiplying the mouth area with the distance towed. Typically, 10 000–60 000 m^3^ of seawater was filtered by a 0–200 m tow. Vertical profiles of temperature, salinity and chlorophyll *a* were collected at all stations (Fig 1B) with a Sea-Bird Electronics SBE 9 CTD. Also, the onset of the phytoplankton spring bloom (OPB) was estimated at all stations using weekly (8-day, 25 x 25 km grid) surface chlorophyll *a* data from satellite images [32]. The OPB was estimated as the first week of the year in which chlorophyll *a* increased by 5% above the annual median value [32, 34].

### Sample analyses

At sea, the zooplankton samples were sorted into main zooplankton groups (krill, cnidarians, amphipods, fish and shrimp) and then weighed to the nearest gram to estimate biomass. Then, the krill catch was divided into two equal parts using a Motoda splitter [35] and preserved for later analysis in the laboratory ashore. One aliquot was preserved in 4% neutralized formaldehyde and the other frozen. However, four samples were too large to be treated in this way. For these samples, ~10% by weight (~1 kg) of the total catch was taken and preserved as described above.

In the laboratory, the frozen samples were thawed and analysed whole or in aliquots (obtained by a Motoda splitter) containing at least 200 individuals under a stereomicroscope. The level of sub-sampling varied according to the abundance of krill, but usually 1/32 part of the sample was analysed. All krill were identified to species, counted and subsequently staged according to the following maturity classifications: (1) juveniles (indeterminate sex); (2) immature males or females; (3) mature males or females; (4) males with spermatophores in ejaculatory ducts or females with spermatophores attached [9, 36–38]. Total length (TL) and carapace length (CL) were measured using a digital caliper or an ocular micrometer on a stereo microscope. TL was measured from the anterior edge of the eye to the tip of the telson, excluding the setae [16]. CL was taken from the base of the eyestalk to the lateral edge of the carapace [39]. In cases where the telson or eyes were damaged, only the CL was measured. For these individuals (4280 individuals of a total of 7552 measured), the TL was estimated using equations obtained by linear regressions between TL and CL for intact individuals (*M*. *norvegica*: TL = 3.100CL+7.166, *r*^2^ = 0.86, n = 694, *P*<0.001; *T*. *inermis*: TL = 2.322CL+9.377, *r*^2^ = 0.67, n = 467, *P*<0.001). Sex ratio (the number of males/number of females) was estimated for *M*. *norvegica* and *T*. *inermis* at stations where at least 25 individuals were sexed.

We did not test specifically for size selectivity of the macrozooplankton trawl in our study. However, the study of Krag et al. [40] who estimated the size selectivity of pelagic trawls for catching Antarctic krill provides some insights. From this study, it may be concluded that a pelagic trawl similar to that used in our study with a mesh size of 6 mm (stretched inside opening), will retain ~95% of krill larger than ~16 mm total length. Based on this we feel confident that at least the adults of both *T*. *inermis* and *M*. *norvegica* were sampled effectively in our study.

### Statistical analyses

Because the spatial distribution of krill was very uneven, the abundance data were ln(x+1) transformed to stabilize the variance before performing statistical analyses. For the same reason, we use the median rather than mean to describe central tendency of abundance. No significant differences were found in the abundance of krill between day and night catches for all species combined and the species separately (*t*-test, 19 day samples, 7 night samples, *P*>0.05). Similarly, no significant differences were found in the length-frequency distributions of *M*. *norvegica* and *T*. *inermis* between the day and night samples (Two-sample Kolmogorov & Smirnov tests, *P*>0.05). Indicating no size-related net avoidance under the different light regimes. Based on these results all samples were treated as comparable. To compare the abundance, distribution, and population structure of *M*. *norvegica* and *T*. *inermis* between the three main hydrographic regions, data were divided into the southwest, north and east regions (Fig 1) [25–27].

Linear and multiple linear regressions were used to explore relationships between the distribution of krill on one hand and temperature (means of 0–200 m), salinity (means of 0–200 m), chlorophyll *a* (means of 0–50 m, mg m^-3^), bottom depth (derived from the ship’s echosounder) and the onset of the spring phytoplankton bloom on the other. Shapiro-Wilk test was used to verify the normality of variables. Chlorophyll *a* and bottom depth were subject to natural logarithmic data transformation to normalize the data. For the multiple linear regression, explanatory variables were tested for collinearity by pairwise scatterplots, Pearson’s correlation coefficients and variance inflation factors (VIF) [41]. Collinear variables with VIF>5 were not used together in the models. Akaike Information Criteria (AIC) was used to select the best stepwise backward multiple regression models [41].

*M*. *norvegica* and *T*. *inermis* cohorts were determined by fitting normal distributions using finite mixture models [42]. The split of the length-frequency into their cohort components was done using the separation index (SI) $SI=\frac{\Delta \mu_{i+1}}{\Delta \sigma_{i+1}}$; where SI>2 was used as the bimodal separation criteria [43, 44]. A similar approach has been used in other studies on krill (e.g. Dalpadado and Skjoldal [45]). Chi-square tests were also used to double check if the cohort components were suitable. The cohort analysis was conducted on TL data, and we only used stations where at least 50 individuals were measured.

Two-sample Kolmogorov & Smirnov tests were used to compare the length-frequency distributions for each species between regions and sexes statistically. In these analyses, only stations with at least 50 length measurements were used. The TL measurements were binned to 1 mm length intervals, and values in each bin were adjusted to reflect the total catch [46], i.e. the numbers in each bin were converted to abundance (individuals 1000 m^-3^).

## Results

### Physical environment

Silva et al. [32] have described the hydrographic and phytoplankton conditions during the survey, and therefore we only give an overview of the main features here (Table 1). In general, temperature and salinity were higher in the southwest than in the north and east. On average, both north and east had lower mean chlorophyll *a* concentrations (0.7 and 0.9 mg m^-3^, respectively) than the southwest (2.4 mg m^-3^). The bloom started between weeks 16 and 23 (i.e. mid-April—early June) in the southwest, between weeks 16 and 26 (i.e. mid-April—mid-June) in the north and between weeks 17 and 28 (i.e. late April—mid-July) in the east. When the survey was conducted (the latter half of May 2013), about two to four weeks had passed since the start of the phytoplankton bloom in the southwest and north, while in the east, the bloom was in the initial phase of development (Table 1). In accordance with these findings, both nitrate and silicate were low in the southwest, the silicate being almost depleted, which suggested that the diatom bloom had already peaked when the sampling took place [47].

**Table 1:** Mean (±SE) environmental conditions in the southwest, north and east of Iceland in late May 2013.

Region	Mean temperature at 50 m (°C)	Mean temperature at 200 m (°C)	Salinity range at 50 m	Salinity range at 200 m	Mean chlorophyll *a* (mg m^-3^, 0–50 m)	OPB range (Weeks)
Southwest	6.2 ± 0.02	6.5 ± 0.2	33.64–35.25	34.48–35.24	2.4 ± 0.04	16–23
North	1.4 ± 0.02	2.2 ± 0.2	34.18–35.06	34.72–35.01	0.7 ± 0.02	16–26
East	0.3 ± 0.01	1.3 ± 0.2	34.58–35.24	34.84–35.22	0.9 ± 0.01	17–28

OPB denotes the onset of the phytoplankton spring bloom (week of the year).

### Distribution and abundance

Four krill species, *M*. *norvegica*, *T*. *inermis*, *T*. *longicaudata* and *Thysanopoda acutifrons*, were found in the samples. However, the latter two species were only found rarely (average values for all stations of 1.4 ± 0.36 and 0.6 ± 0.3 individuals 1000 m^-3^, respectively) and are therefore not considered further in our analysis.

**Fig 2:**
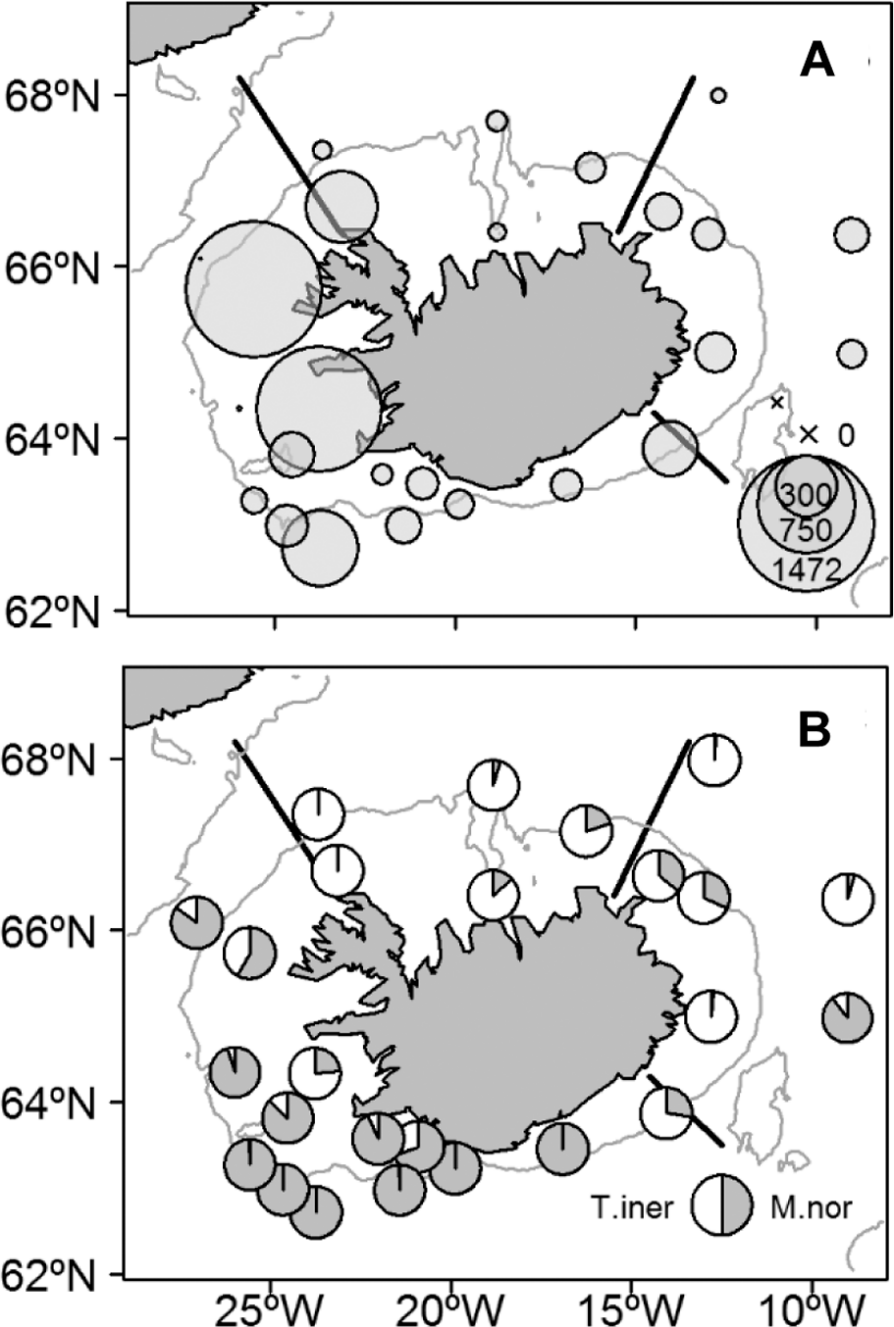
Distribution of *Meganyctiphanes* and *Thysanoessa inermis* around Iceland, 14–29 May 2013. (A) Combined abundance of *M*. *norvegica* and *T*. *inermis* (individuals 1000 m^-3^). (B) Relative abundance of *M*. *norvegica* (M.nor) and *T*. *inermis* (T.iner). The grey line indicates the 400 m isobath.

**Fig 3:**
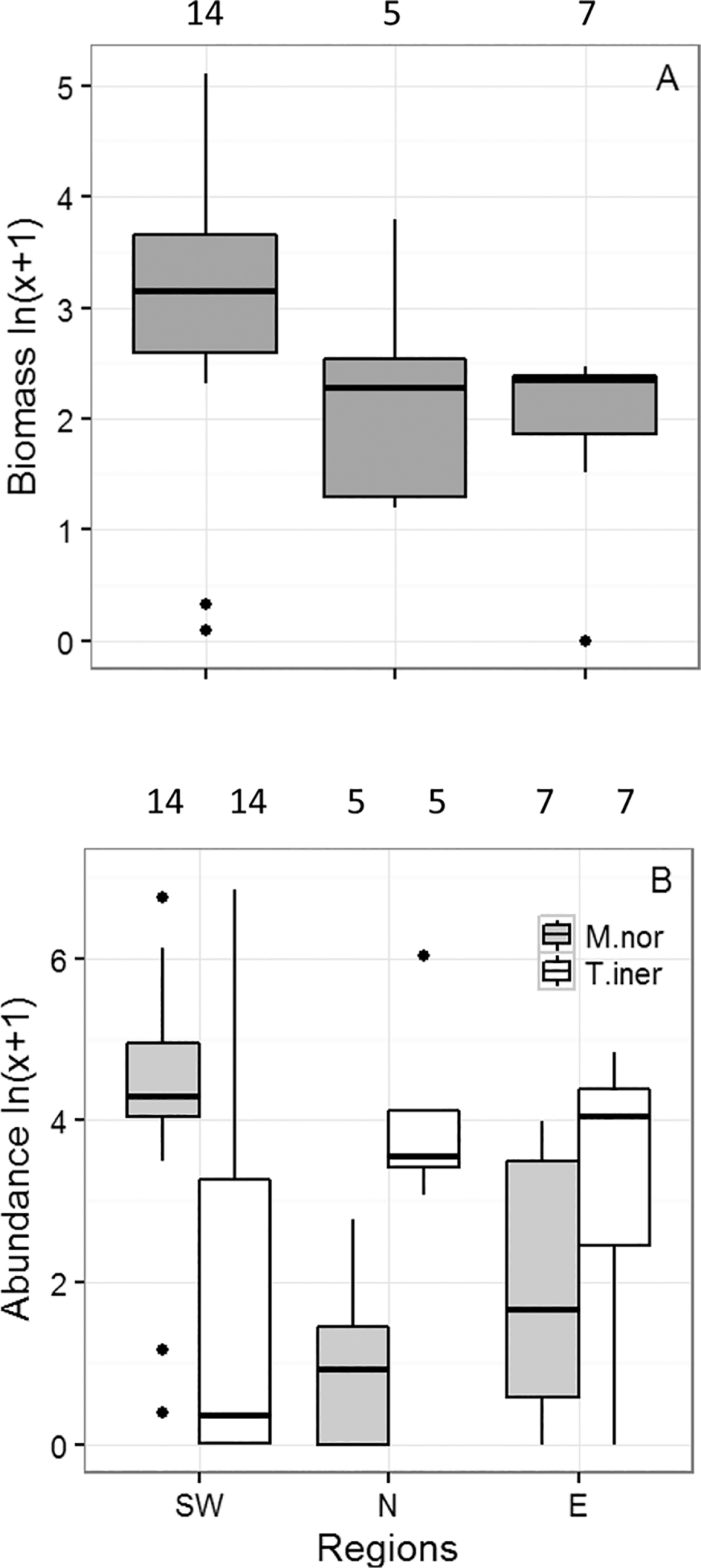
Box-Whisker plots of biomass and abundance of krill divided into stations taken southwest (SW), north (N) and east (E) of Iceland, 14–29 May 2013. (A) Biomass of *Meganyctiphanes*. *norvegica* and *Thysanoessa inermis* combined (ln (g wet weight 1000 m^-3^ + 1)). (B) Abundance (ln (individuals 1000 m^-3^ + 1)) of *M*. *norvegica* and *T*. *inermis* separately. Vertical lines give the total range; horizontal lines indicate the median values and boxes show the range of 50% of all data (between upper and lower quartiles). Dots represent outliers. The number of samples (stations) for each region are shown along the top of the panels.

Abundance (individuals 1000 m^-3^) of krill (both species combined) was very unevenly distributed (Fig 2). Values were generally highest in the southwest and lowest in the north and east (Figs 2 and 3). In the southwest, *M*. *norvegica* was relatively much more abundant than *T*. *inermis*. The opposite was true in north and east (Fig 2B).

Biomass of both species combined was significantly higher in southwest (~22.4 g 1000 m^-3^) than in north (~8.8 g 1000 m^-3^) and east (~9.4 g 1000 m^-3^) (Kruskal-Wallis with Dunn’s post hoc, *χ*^*2*^ = 6.14, n = 14, 5, and 7 for southwest, north and east, respectively, *P*<0.05) (Fig 3), while no significant differences were found between biomasses in the north and east (Kruskal-Wallis with Dunn’s post hoc, n = 5, and 7 for north and east, respectively, *P* = 0.29). Reflecting this, the abundance of *M*. *norvegica* was higher in the southwest (~72.9 individuals 1000 m^-3^) than in north (~1.5 individuals 1000 m^-3^) and east (~4.3 individuals 1000 m^-3^) (Kruskal-Wallis with Dunn’s post hoc, *χ*^*2*^ = 12.74, n = 14, 5, and 7 for southwest, north and east, respectively, *P*<0.001). However, no significant differences were found in the abundance of *T*. *inermis* between any of the regions (Kruskal-Wallis with Dunn’s post hoc, *χ*^*2*^ = 3.63, n = 14, 5, and 7 for southwest, north and east, respectively, *P*>0.05).

### Sex ratio, maturation and spawning

**Fig 4:**
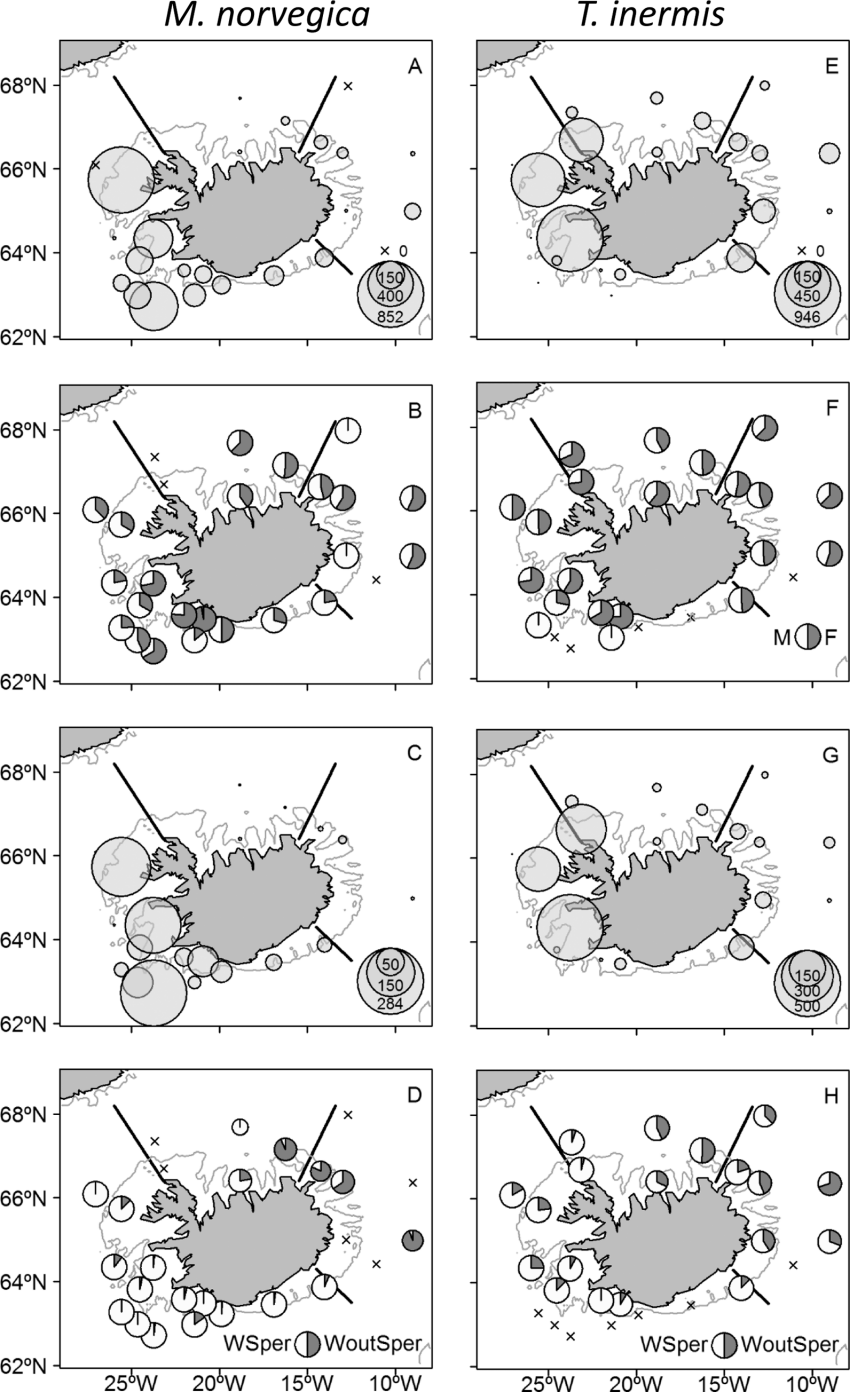
Distribution of abundance, sex ratio, maturation and spawning activity of *Meganyctiphanes norvegica* and *Thysanoessa inermis* around Iceland, 14–29 May 2013. (A, E) Abundance (individuals 1000 m^-3^), (B, F) sex ratio, (C, G) abundance of females with spermatophores (individuals 1000 m^-3^), and (D, H) relative abundance of females with (WSper) and without (WoutSper) spermatophores. Grey line is the 400 m isobath. In B and F, the crosses indicate stations where no individuals were found.

The sex ratio (male/female) of both species was highly variable (Fig 4B and 4F). For *T*. *inermis* the sex ratio was similar in all regions (~0.8; Pearson’s χ^2^ = 4.8, n = 10, 5, and 6 for southwest, north and east, respectively, *P*>0.05). For *M*. *norvegica*, a statistical test could not be carried out due to too few samples (n = 14, 2, and 3 for southwest, north and east, respectively).

For both species, the abundance of females with spermatophores was generally higher in the southwest than in north and east (Fig 4C and 4G). For *M*. *norvegica*, the percentage of females with spermatophores was also higher in the southwest than in north and east, whereas for *T*. *inermis*, the percentage of females with spermatophores was more similar in all regions (Fig 4H).

**Fig 5:**
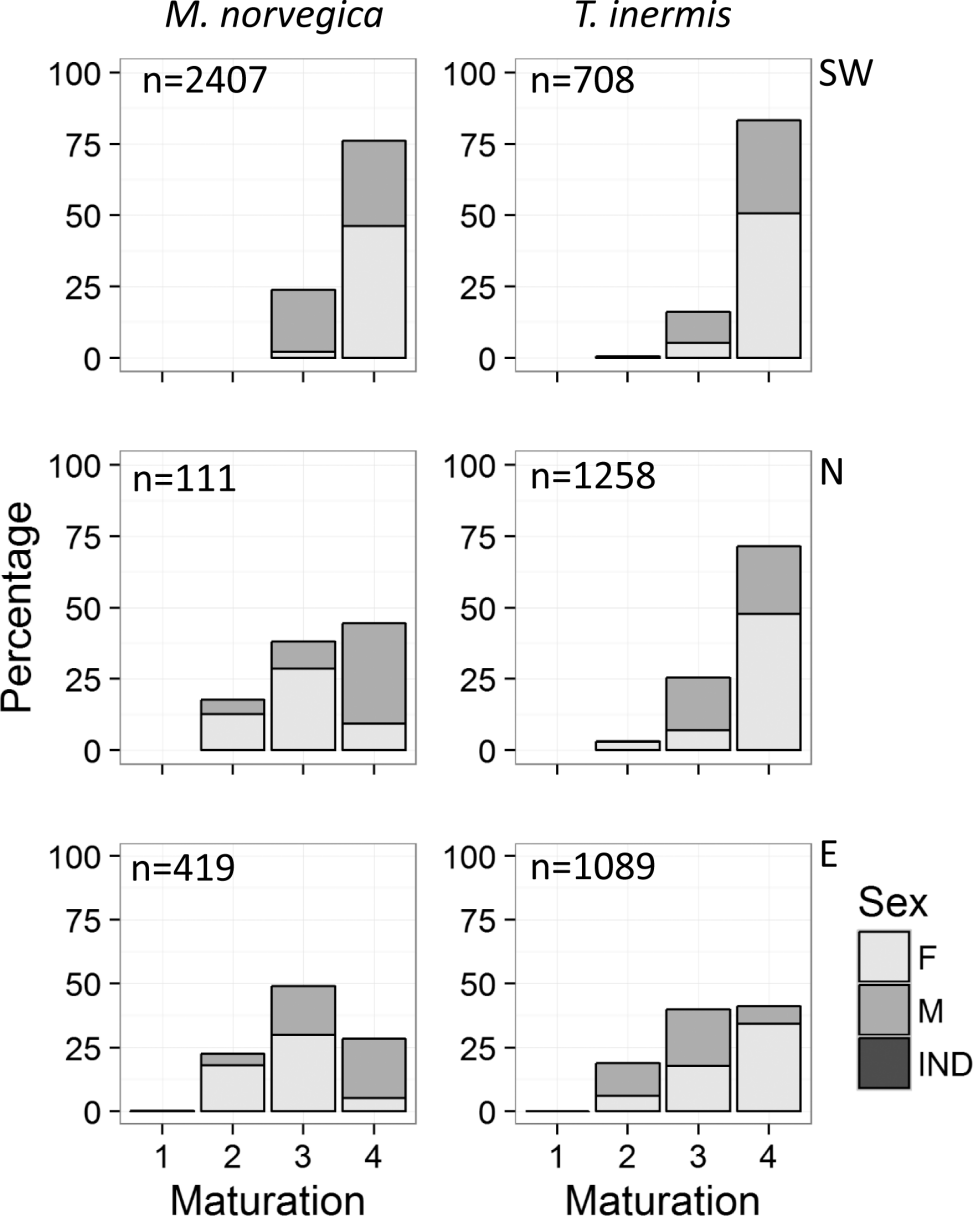
Sex and maturity stages of *Meganyctiphanes norvegica* and *Thysanoessa inermis* in the southwest (SW), north (N) and east (E) of Iceland, 14–29 May 2013. F: females, M: males and IND: Indeterminate sex. The number of individuals analysed is stated in each graph. See Materials and Methods for details on the maturation stages.

In the southwest, most of *M*. *norvegica* were females bearing spermatophores (stage 4, 46%), or males with spermatophores in their ejaculatory ducts (stage 4, 30%) (Fig 5), and no juveniles (stage 1) or immature individuals (stage 2) were found. In the north and east, the proportion of animals with spermatophores (stage 4) was lower and proportion of immature (stage 2) and mature (stage 3) animals higher. The relatively low percentage of females with spermatophores to the north and east of Iceland possibly indicates relatively low breeding activity of animals in these regions at the time of sampling. However, this finding could also reflect population mixing or slower development in the colder waters.

Maturation of *T*. *inermis* females and males was more advanced in the southwest and north than in the east, as indicated by the relatively high proportion of individuals of both sexes at stage 4 in the southwest and north (Fig 5). In the east, the percentage of females of *T*. *inermis* with spermatophores (stage 4) was much lower (34%) than in the southwest (51%) and north (48%). Similarly, the proportion of males with spermatophores (stage 4) was much lower in east (7%) than in southwest (33%) and north (24%). As found for *M*. *norvegica*, juveniles (stage 1, 0.1%,) and immatures (stage 2, 19%,) of *T*. *inermis* were most abundant in the east.

### Factors affecting distribution

**Fig 6:**
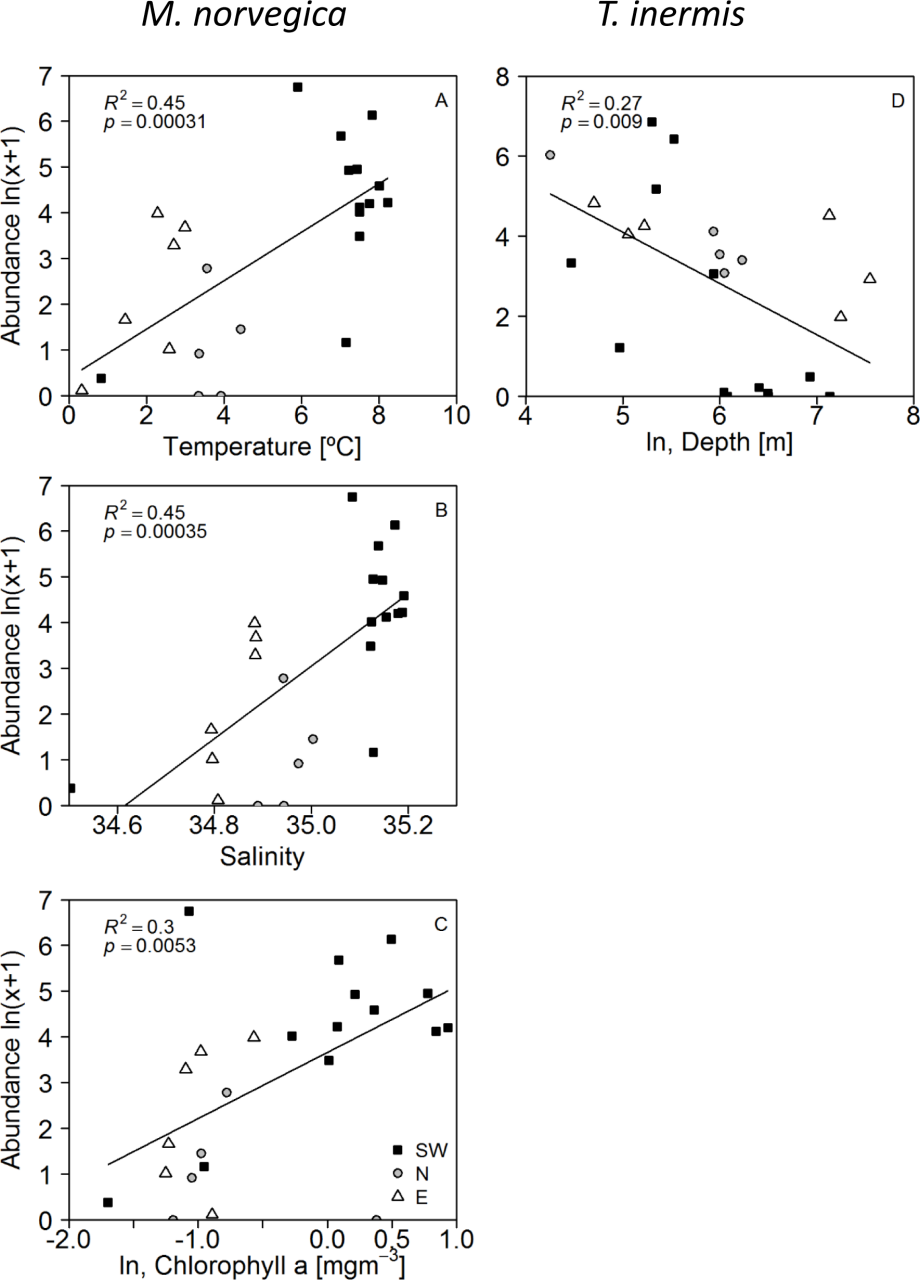
Relationships between the abundance of *Meganyctiphanes norvegica* and *Thysanoessa inermis* and environmental variables. (A) Temperature (°C, average 0–200 m), (B) salinity (average 0–200 m), (C) chlorophyll *a* (mg m^-3^, 0–50 m) and (D) bottom depth (n = 24 for all regressions). The shading of the dots indicates if the data are from the southwest (SW: black squares), north (N: grey circles) and east (E: white triangles) of Iceland.

Simple linear regressions showed that the abundance of *M*. *norvegica* was positively related to temperature, salinity and chlorophyll *a* while the abundance of *T*. *inermis* was negatively related to bottom depth (Table 2 and Fig 6).

**Table 2:** Pearson’s correlation matrix (n = 24).

	OPB	T	S	D	Chl *a*	*M*. *nor*
Temperature	-0.14					
Salinity	-0.27	0.93***				
Depth	-0.38	-0.18	-0.1			
Chl *a*	-0.22	0.77***	0.74***	-0.19		
*M*. *norvegica*	-0.17	0.67***	0.67***	-0.01	0.55**	
*T*. *inermis*	0.39	-0.29	-0.16	-0.52**	-0.13	-0.04

Variables are the onset of the phytoplankton spring bloom (OPB), temperature (°C, average 0–200m), salinity (average 0–200 m), bottom depth (ln-transformed, m), chlorophyll *a* (ln-transformed, mg m^-3^, average 0–50 m) and abundance of *Meganyctiphanes norvegica* and *Thysanoessa inermis* (ln(x+1)). Asterisks indicate significance level

*** *P* <0.001 and

** *P*<0.01.

It is reasonable to assume that the environmental variables may act together in affecting the distribution of *M*. *norvegica* and *T*. *inermis*, and therefore multiple linear regression analyses were attempted. Temperature and salinity were highly correlated (Pearson’s *r* = 0.93, n = 26, *P*<0.0001; VIF>10), accordingly models were run with these variables separately. The analyses showed that the abundance of *M*. *norvegica* was positively related to temperature (*r*^*2*^ = 0.45, n = 24, Table 3), whereas, the abundance of *T*. *inermis* was negatively related to both temperature and bottom depth (*r*^*2*^ = 0.42, n = 24, Table 3, model 11).

**Table 3:** Results of stepwise multiple linear regression analyses carried out to predict the abundance of *Meganyctiphanes norvegica* and *Thysanoessa inermis* (n = 24).

Species	Model equation	Model	Variables removed	*r*^2^	*P*	AIC
*M*. *nor*	1	ln(M.nor+1) = -0.19+0.21T*-0.007OPB +0.24ln(Chl)+0.25ln(D)	S	0.47	0.013	58.32
	2	ln(M.nor+1) = -0.39+ 0.21T*+ 0.26ln(Chl)+0.27ln(D)	S, OPB	0.47	0.005	56.34
	3	ln(M.nor+1) = -0.53+0.24T***+0.26ln(D)	S, OPB, Chl *a*	0.47	0.001	54.52
	4	ln(M.nor+1) = 0.18+0.23T***	S, OPB, Chl *a*, D	0.45	0.0003	**53.08**
	5	ln(M.nor+1) = -105+2.99S*+0.024OPB+0.41ln(Chl)+0.24ln(D)	T	0.46	0.014	58.62
	6	ln(M.nor+1) = -101 + 2.92S* + 0.38ln(Chl) +0.18ln(D)	T, OPB	0.46	0.005	56.77
	7	ln(M.nor+1) = -102+2.96S*+0.33ln(Chl)	T, OPB, D	0.45	0.002	55.02
	8	ln(M.nor+1) = -119+3.43S***	T, OPB, D, Chl *a*	0.45	0.0003	53.34
*T*. *iner*	9	ln(T.iner+1) = 4.1–0.19T+0.07OPB+0.58ln(Chl)-1.24ln(D)*	S	0.46	0.017	62.62
	10	ln(T.iner+1) = 4.1–0.13T+0.06OPB-1.30ln(D)*	S, Chl *a*	0.44	0.008	61.33
	11	ln(T.iner+1) = 5.7–0.14T*-1.45ln(D)**	S, Chl *a*, OPB	0.42	0.003	**60.05**
	12	ln(T.iner+1) = 14.2–0.32S+0.06OPB-0.48ln(Chl)-1.21ln(D)*	T	0.35	0.073	66.90
	13	ln(T.iner+1) = 33.8–0.88S+0.07OPB-1.16ln(D)*	T, Chl *a*	0.34	0.037	65.24
	14	ln(T.iner+1) = 2.19+0.09OPB-1.06ln(D)*	T, Chl *a*, S	0.32	0.018	64.04
	15	ln(T.iner+1) = 4.55–1.28ln(D)*	T, Chl *a*, S, OPB	0.27	0.009	63.59

Temperature (T, 0–200 m), salinity (S, 0–200 m), bottom depth (D, m), the onset of the phytoplankton spring bloom (OPB, weeks) and chlorophyll *a* (mg m^-3^, 0–50 m) were used as independent variables. For each model, regression coefficients, the total variance explained (*r*^2^), significance (*P*) and Akaike Information Criteria (AIC) are given. The AIC values in bold indicate the best model for *M*. *norvegica* and *T*. *inermis*. Asterisks indicate significance level

*** *P* <0.001

** *P*<0.01 and

* *P*<0.05.

### Length-frequency distribution and population structure

**Fig 7:**
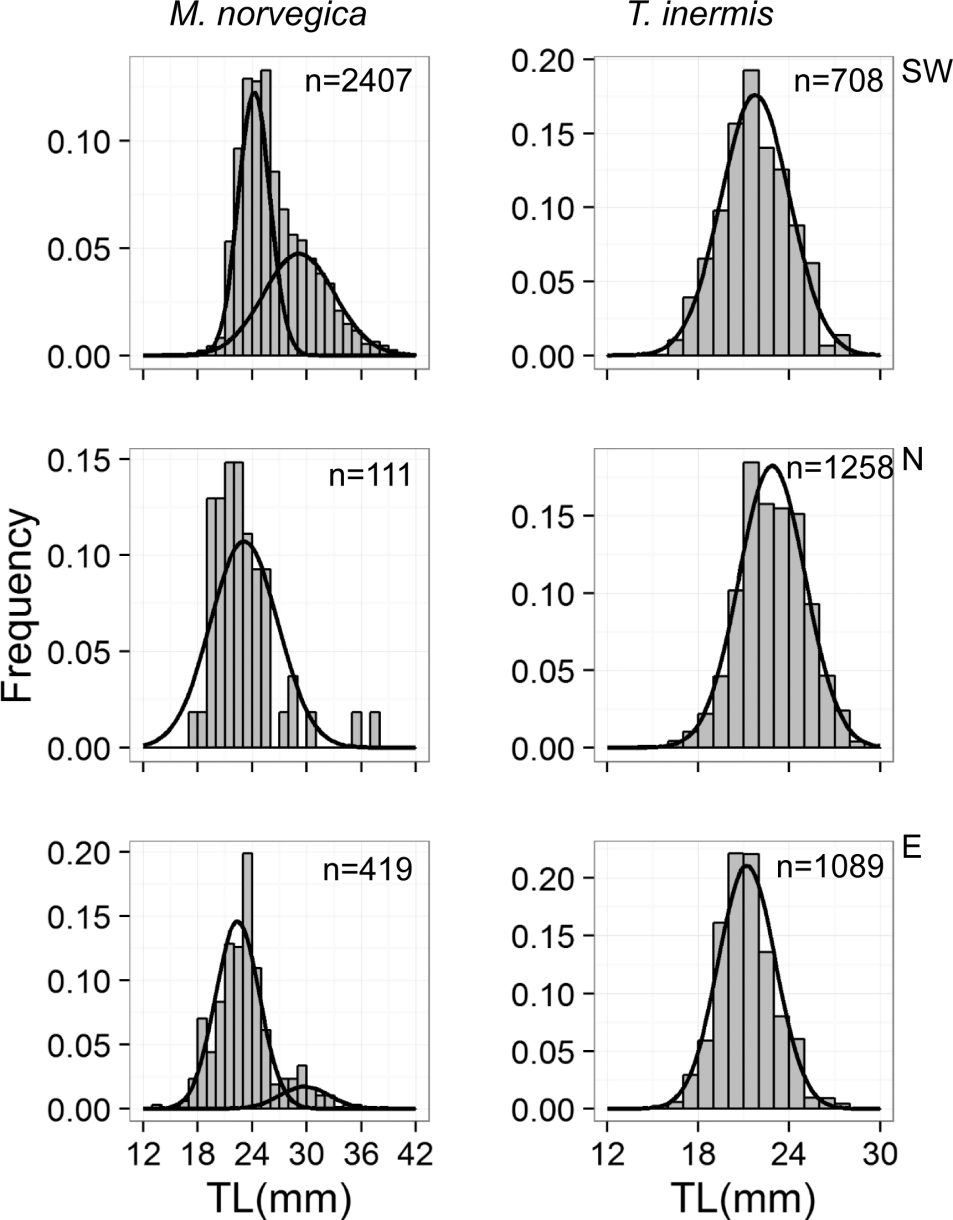
Length-frequency distributions of *Meganyctiphanes norvegica* and *Thysanoessa inermis* in the southwest (SW), north (N) and east (E) of Iceland, 14–29 May 2013. The curves show the cohort components for each region and species. The number of individuals measured is stated in each graph. Note that the axes have different scales. See the Materials and Methods for an explanation how the cohorts were identified.

Finite mixture models were used to identify the cohorts statistically in each region (Fig 7). The modelled population structure of *M*. *norvegica* was bimodal in the southwest and east, probably reflecting 2-year classes. However, the test failed to identify two length modes in the north (χ^2^ = 7e^-09^, *P*>0.05), but this may have resulted from the few number of samples. The population structure was unimodal for *T*. *inermis* in all three regions. For all regions, the 1 year class of *M*. *norvegica* had much higher frequency than the 2 year class.

For *M*. *norvegica*, the smaller length mode (1 year old individuals) had a mean length of 24.2 mm in the southwest and ~22.0 mm in north and east (Fig 7). The larger length mode (2 year old) of *M*. *norvegica* had an average length of 29.1 mm in the southwest and 29.7 mm in the east. For *T*. *inermis*, the single length mode was larger in the north (22.9 mm) than in the southwest (21.8 mm) and east (21.2 mm).

Two-sample Kolmogorov & Smirnov tests were used to examine if the length-frequency distributions of *M*. *norvegica* and *T*. *inermis* varied among regions for all stages combined and for the sexes separately. For *M*. *norvegica*, the tests showed that the length-frequency distributions were similar between regions for all stages combined (n = 2407, 111 and 419 for southwest, north and east, respectively, *P* >0.05), and also for males and females separately for southwest (n = 1272 and 975 for males and females, respectively, *P*>0.05), north (n = 34 and 30 for males and females, respectively, *P* >0.05) and east (n = 152 and 188 for males and females, respectively, *P*>0.05).

**Fig 8:**
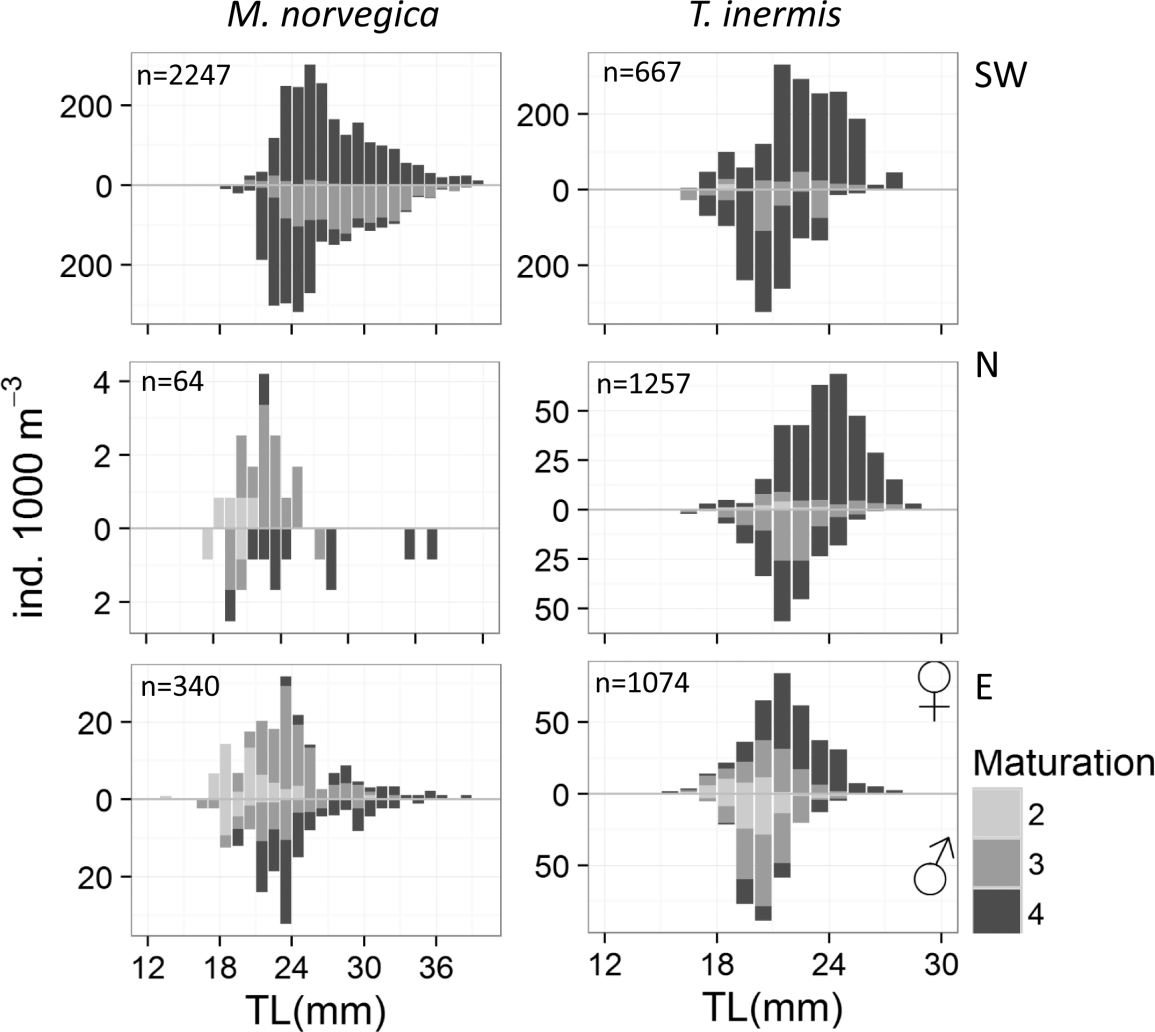
Length-frequency distributions of *Meganyctiphanes norvegica* and *Thysanoessa inermis* in the southwest (SW), north (N) and east (E) of Iceland divided by sex and maturation stages, 14–29 May 2013. Bars above abscissas indicate females and below are the males. The number of individuals measured is stated in each graph. Note that the axes have different scales. See Materials and Methods for details on the maturation stages.

For *T*. *inermis*, the length-frequency distributions were significantly different between north and east (n = 1257 and 1074 for north and east, respectively, *P* = 0.02) but similar among the other regions (n = 667, 1257 and 1074 for southwest, north and east, respectively, *P*>0.05). Males and females of *T*. *inermis* had similar length distributions in the southwest (n = 302 and 365 for males and females, respectively, *P*>0.05). However, significant differences were found in the length distributions of *T*. *inermis* males and females in north (n = 522 and 735 for males and females, respectively, *P* = 0.05) and east (n = 491 and 583 for males and females, respectively, *P* = 0.05), with the females being on average larger than the males (Fig 8).

In the southwest, almost all female and most male *M*. *norvegica* 1 year olds (the smaller length mode) were with spermatophores (stage 4), indicating that the populations in these areas reach maturity and reproduce as 1 year olds. Conversely, in the north and east, only a relatively small part of the 1 year old females were carrying spermatophores, whereas the 2 year olds (the larger length mode) in the east were comprised solely of mature individuals (stage 3), and most with spermatophores (stage 4), indicating that in these areas most of the populations will reproduce as 2 year olds.

In southwest and north the single length mode of *T*. *inermis* was made up of mature individuals (stage 3) and most with spermatophores (stage 4), indicating spawning activity in these regions. In the east, the population structure was more mixed consisting of stages 2–4. However, in the east most of the females were bearing spermatophores (stage 4), showing that the population was also reproducing there.

## Discussion

### Sampling considerations

When interpreting the present data, it is important to bear in mind that due to the rather large meshes of the pelagic trawl (mesh size of 4 mm, 6 mm stretched), some of the juveniles will inevitably escape. Thus, as discussed in the Methods section, it is likely that the trawl used for sampling will not catch effectively krill <16 mm total length. Accordingly, the juveniles of *T*. *inermis* (total length ~12–20 mm in May, [9–12]) were not sampled effectively, while those of *M*. *norvegica* (~16–20 mm in May, [9, 13]) probably were. In spite of these limitations, we nevertheless believe that the present material demonstrates the main patterns in distribution and population structure of these two species in the waters around Iceland during spring.

Further, the fact that we only sampled down to 200 m depth, whereas krill may stay as deep as ~100–500 m during spring and summer [9, 16, 17], may represent a problem. However, at most of the sampling stations the bottom depth was <200 m, meaning that at these stations the whole population was sampled by the 0–200 m trawl sampling. At a few offshore stations (8 in all), however, the bottom depth was greater than 200 m, and this would possibly have led to underestimates of krill abundance at these stations. In order to explore the depth distribution of krill at these stations, we have analysed acoustic data that were collected along the survey routes using Simrad EK601 scientific echo sounders (Kongsberg Maritime AS) with 38 and 120 kHz transducers allowing the backscatter frequency response of euphausiids down to 300 m to be observed (see Reynisson and Gislason [48] for further details). This analysis showed that krill were mostly distributed above 200 m depth both in the onshore and offshore areas. Whilst it cannot be discounted that some krill stayed below the sampling depth of the acoustics (300 m) at the deepest offshore stations, we feel that these results nevertheless support the interpretation that we were catching all or the main part of the populations in the deep offshore areas also.

Moreover, while the temporal coverage of this study may not be optimal, krill tends to be patchy distributed [12] making it difficult to obtain representative samples of the whole populations. However, the large-scale nature of this study and the fact that we combined samples by oceanographic regimes, at least partly remedies these limitations.

### Distribution

Our results show a high degree of spatial variability in the distribution of both *M*. *norvegica* and *T*. *inermis* around Iceland (Fig 4A and 4E). Both species occurred in highest abundances in the southwest, whereas *T*. *inermis* was also relatively abundant in north and east (Figs 2C, 3B, 4A and 4E). The multiple linear regression analysis showed that the abundance of *M*. *norvegica* was positively related to temperature, whereas *T*. *inermis* was negatively related to temperature and bottom depth (Table 3). Therefore, the different distribution patterns of *M*. *norvegica* and *T*. *inermis* is most likely determined by the distribution of different water masses around Iceland and topography. The negative association of *T*. *inermis* with bottom depth is in agreement with other studies that have shown *T*. *inermis* to be mainly confined to shelf areas and the coastal banks [9, 11, 12, 14, 49]. Our findings are in agreement with the generalization that *T*. *inermis* is abundant in Subarctic–Arctic coastal regions while *M*. *norvegica* is mostly found in regions where Atlantic water predominates [11,49, 50].

Previous studies have shown both *M*. *norvegica* and *T*. *inermis* to be opportunistic in their feeding behaviours, with the trophic position of both species being dependent on the availability of food [51–55]. Thus, both species are able to supplement herbivorous feeding by omnivory or carnivory during periods of low phytoplankton growth. Studies have shown that *T*. *inermis* fed on larger diatom cells, detritus and small zooplankton [52, 55]. The preference for smaller zooplankton may be a factor in explaining the more on-shelf distribution pattern of this species as compared to *M*. *norvegica*.

### Maturity

The numbers of reproductively active females of both species (i.e. females with spermatophores) were highest in the southwest (Fig 4C and 4G) which fits with that we found the highest number of eggs and larvae in the same areas during the survey [32]. The high breeding activity of *M*. *norvegica* in southwest accords well with the findings of Einarsson [9] found. However, we also observed a relatively high percentage (~40–60%) of female *M*. *norvegica* carrying spermatophores in the north indicating some breeding activity also there (Fig 4D). However, these active reproductive animals may well have been brought to the northern regions from the south by currents (Fig 1).

The distribution of *T*. *inermis* females carrying spermatophores was mainly restricted to the coastal banks in the southwest thus indicating high breeding activity there (Fig 4G). In the north and east, breeding activity appeared much more limited as deduced from the number and percentage of females with spermatophores (Fig 4G and 4H). This is at odds with Einarsson [9], in that high breeding activity of *T*. *inermis* occurs to the north and east of Iceland.

The sex ratios of *M*. *norvegica* were different between the three regions, with a higher proportion of males in the southwest (1.3) than in the north (1.1) and east (0.8). Mauchline and Fisher [16] suggested that sex ratio changes in a population could be used to detect mortality at breeding as mortality rates can vary between sexes during the breeding period [56], with studies indicating that males die after spermatophore transfer and females die after egg-laying [16, 50]. If this holds true, then the higher proportion of males in southwest compared to the other regions could indicate earlier breeding activity in southwest relative to the other regions.

The sex ratios of *T*. *inermis* were similar all around Iceland, with generally higher proportions of females than males in the samples (~0.8). Similarly, in the Barents Sea and Balsfjorden, northern Norway, the sex ratio of *T*. *inermis* tends to oscillate close to one throughout the year [50, 57].

### Population structure

The modelled length-frequency distributions of *M*. *norvegica* were bimodal in the south and east indicating a 2 year life cycle. In the north the two length modes were not statistically significant probably because of too low sample size (Fig 7). However, the distribution of maturity stages indicates that the population reaches maturity as 1 year olds in all areas (Fig 8). Similarly, previous studies from around Iceland and in the Irminger Sea [9, 13], found *M*. *norvegica* having a life span of 2 years and reaching maturity at 1 year (Fig 8).

In contrast, we found that the modelled length-frequency distributions were unimodal for *T*. *inermis* (Fig 7). Einarsson [9] found that populations of *T*. *inermis* in the north and east reached maturity when 2 year old, while in the south a part of the population had a life expectancy of only 1 year. Saunders et al. [12] found during spring a unimodal length distribution of *T*. *inermis* in the Irminger Sea consisting of 2 year old individuals. Similarly, Astthorsson [10] and Astthorsson and Gislason [11] found that the 1 and 2 year old year classes of *T*. *inermis* tend to overlap in size in May in Icelandic waters. Therefore, the unimodal length distribution of *T*. *inermis* in our study may represent 2 year classes (1 and 2 year olds) merged into one size mode.

The fact that there was no sign of the 0 year class for neither species in our study (Fig 7) is likely because both species are <10 mm in May-June as a 0 year class [9]. Therefore, the 0 year class was not caught effectively by the 4x4 mm meshes of the macrozooplankton trawl. Similarly, the larval survey indicated low abundance of furcilia and juveniles at this time of the year [32].

In the southwest and north, *T*. *inermis* were larger (~22 and 23 mm) than in the east (~21 mm, Fig 8). It is well established that the warmer waters off the southwest coast of Iceland are more productive than the colder areas in north and east [8, 27, 30]. The influence of warm Atlantic water in the southwest and north (Table 1) may thus promote faster growth of *T*. *inermis*, as temperature and food conditions are known to affect the maturation and growth of krill [10, 13, 39].

Females of *T*. *inermis* tended to be larger than the males (Fig 8). This most likely reflects either different growth rates of males and females with the females growing faster than the males or different death rates of males and females with older and larger males dying off from the populations at a faster rate than the females. The same gender-related variability in the size of *T*. *inermis* females and males was also found by Astthorsson [10] in Ísafjord-deep, northwest of Iceland but was not detected in the subarctic waters north of Iceland [11]. In the Barents Sea, Dalpadado and Ikeda [58] found no difference in the growth of *T*. *inermis* females and males during spring.

### Conclusions

This is the most comprehensive study of variation in euphausiids abundance and population dynamics in relation to the underlying oceanographic regime in Icelandic waters to date. Our study showed that hydrographic parameters, particularly temperature and underlying bathymetry, had a major role in influencing the distribution, reproductive behaviour and population structure of two key species, *M*. *norvegica* and *T*. *inermis*, in this important ecosystem. The influence of warm and productive Atlantic water, in particular, appeared to be important in the development and life cycles of these species in this region. Our data provide important contemporary baselines for ongoing ecosystem and food web studies in this region, and our results highlight the requirement for elucidating the environmental mechanisms influencing the distribution and ecology of krill species around Iceland to underpin effective ecosystem-based management strategies in the region.

## References

[1] Gislason A, Astthorsson OS. Seasonal cycle of zooplankton southwest of Iceland. J Plankton Res. 1995; 17: 1959–1976. doi: 10.1093/plankt/17.10.1959

[2] Astthorsson OS, Pálsson OK. Predation on euphausiids by cod, *Gadus morhua*, in winter in Icelandic subarctic waters. Mar Biol. 1987; 96: 327–334. doi: 10.1007/BF00412513

[3] Dommasnes A, Melle W, Dalpadado P, Ellertsen B. Herring as a major consumer in the Norwegian Sea. ICES J Mar Sci. 2004; 61: 739–751. doi: 10.1016/j.icesjms.2004.04.001

[4] Jaworski A, Ragnarsson SÁ. Feeding habits of demersal fish in Icelandic waters: a multivariate approach. ICES J Mar Sci. 2006; 63: 1682–1694. doi: 10.1016/j.icesjms.2006.07.003

[5] Pálsson ÓK, Bjӧrnsson H. Long-term changes in trophic patterns of Iceland cod and linkages to main prey stock sizes. ICES J Mar Sci. 2011; 68: 1488–1499. doi: 10.1093/icesjms/fsr057

[6] Sigurjónsson J, Víkingsson GA. Seasonal abundance of and estimated food consumption by cetaceans in Icelandic and adjacent waters. J Northw Atl Fish Sci. 1997; 22: 271–287. doi: 10.2960/J.v22.a20

[7] Astthorsson OS, Gislason A. On the food of capelin in the subarctic waters north of Iceland. Sarsia. 1997; 82: 81–86. doi: 10.1080/00364827.1997.10413641

[8] Astthorsson OS, Gislason A, Jónsson S. Climate variability and the Icelandic marine ecosystem. Deep-Sea Res II. 2007; 54: 2456–2477. doi: 10.1016/j.dsr2.2007.07.030

[9] Einarsson H. Euphausiacea I. Northern Atlantic species. Copenhagen: Bianco Luno; 1945.

[10] Astthorsson OS. Ecology of the euphausiids *Thysanoessa raschi*, *T*. *inermis* and *Meganyctiphanes norvegica* in Ísafjord-deep, northwest-Iceland. Mar Biol. 1990; 107: 147–157. doi: 10.1007/BF01313252.

[11] Astthorsson OS, Gislason A. Biology of euphausiids in the subarctic waters north of Iceland. Mar Biol. 1997; 129: 319–330. doi: 10.1007/s002270050172

[12] Saunders R, Rasmussen J, Tarling G, Brierley A. Distribution, population dynamics and growth rates of *Thysanopoda acutifrons*, *Thysanoessa inermis* and *Nematobrachion boӧpis* in the Irminger Sea, North Atlantic. J Mar Biol Assoc UK. 2013; 93: 1287–1301. doi: 10.1017/S0025315412001385

[13] Saunders RA, Ingvarsdottir A, Rasmussen J, Hay SJ, Brierley AS. Regional variation in distribution pattern, population structure and growth rates of *Meganyctiphanes norvegica* and *Thysanoessa longicaudata* in the Irminger Sea, North Atlantic. Prog Oceanogr. 2007; 72: 313–342. doi: 10.1016/j.pocean.2006.09.005

[14] Lindley JA, Williams R. Plankton of the fladen ground during FLEX 76 II. Population Dynamics and Production of *Thysanoessa inermis* (Crustacea: Euphausiacea). Mar Biol. 1980; 57: 79–86. doi: 10.1007/BF00387373

[15] Lindley JA. Population dynamics and production of euphausiids. III. *Meganyctiphanes norvegica* and *Nyctiphanes couchi* in the North Atlantic Ocean and the North Sea. Mar Biol. 1982; 66: 37–46.

[16] Mauchline J, Fisher LR. The biology of euphausiids Russell Frederick S. Sir and Yonge Sir Maurice, editor London and New York: Academic Press; 1969 doi: 10.1016/S0065-2881(08)60468-X

[17] Letessier TB, Falkenhaug T, Debes H, Bergstad OA, Brierley AS. Abundance patterns and species assemblages of euphausiids associated with the Mid-Atlantic Ridge, North Atlantic. J Plankton Res. 2011; 33: 1510–1525. doi: 10.1093/plankt/fbr056

[18] Paulsen O. Plankton investigations in the waters round Iceland and in the North Atlantic in 1904. Meddelelser Fra Kommissionen for Havundersogelser (ser: plankton). 1909; I: 1–56.

[19] Stephensen K. Euphausiacea, Mysidacea, Cumacea, and Nebaliacea. Zool Iceland. 1938; 3: 1–24.

[20] Hansen B, Østerhus S. North Atlantic-Nordic seas exchanges. Prog Oceanogr. 2000; 45: 109–208. doi: 10.1016/S0079-6611(99)00052-X

[21] Malmberg S-A, Kristmannsson S. Hydrographic conditions in Icelandic waters, 1980–1989. ICES Mar Sci Sym. 1992; 195 pp. 76–92.

[22] Stefansson U. North Icelandic waters. Rit Fiskideildar. 1962; 3: 1–269.

[23] Valdimarsson H, Astthorsson OS, Palsson J. Hydrographic variability in Icelandic waters during recent decades and related changes in distribution of some fish species. ICES J Mar Sci. 2012; 69: 816–825. doi: 10.1093/icesjms/fss027

[24] Jónsson S, Valdimarsson H. The flow of Atlantic water to the North Icelandic Shelf and its relation to the drift of cod larvae. ICES J Mar Sci. 2005; 62: 1350–1359. doi: 10.1016/j.icesjms.2005.05.003

[25] Jónsson S. Volume flux and fresh water transport associated with the East Icelandic Current. Prog Oceanogr. 2007; 73: 231–241. doi: 10.1016/j.pocean.2006.11.003

[26] Logemann K, Ólafsson J, Snorrason Á, Valdimarsson H, Marteinsdóttir G. The circulation of Icelandic waters-a modelling study. Ocean Sci. 2013; 10: 763–824. doi: 10.5194/os-9-931-2013

[27] Gislason A. Seasonal and spatial variability in egg production and biomass of *Calanus finmarchicus* around Iceland. Mar Ecol Prog Ser. 2005; 286: 177–192. doi: 10.3354/meps286177

[28] Beare DJ, Gislason A, Astthorsson OS, Mckenzie E. Assessing long-term changes in early summer zooplankton communities around Iceland. ICES J Mar Sci. 2000; 57: 1545–1561. doi: 10.1006/jmsc.2000.0973

[29] Astthorsson OS, Gislason A. Long-term changes in zooplankton biomass in Icelandic waters in spring. ICES J Mar Sci. 1995; 52: 657–668. doi: 10.1016/1054-3139(95)80079-4

[30] Gudmundsson K. Long-term variation in phytoplankton productivity during spring in Icelandic waters. ICES J Mar Sci. 1998; 55: 635–643. doi: 10.1006/jmsc.1998.0391

[31] Gislason A, Astthorsson OS. Distribution patterns of zooplankton communities around Iceland in spring. Sarsia. 2004; 89: 467–477. doi: 10.1080/00364820410009256

[32] Silva T, Gislason A, Astthorsson OS, Marteinsdottir G. Abundance and distribution of early life stages of krill around Iceland during spring. Mar Biol Res. 2016 doi: 10.1080/17451000.2016.1210808

[33] Valdimarsson H, Malmberg S-A. Near-surface circulation in Icelandic waters derived from satellite tracked drifters. Rit Fiskideildar. 1999; 16: 23–39.

[34] Silva T, Gislason A, Licandro P, Marteinsdóttir G, Ferreira ASA, Gudmundsson K et al Long-term changes of euphausiids in shelf and oceanic habitats southwest, south and southeast of Iceland. J Plankton Res. 2014; 36: 1262–1278. doi: 10.1093/plankt/fbu050

[35] Motoda S. Devices of simple plankton apparatus. Memoirs. Faculty of Fisheries. 1959; 7: 73–94.

[36] Cuzin-Roudy J, Amsler MO. Ovarian development and sexual maturity staging in Antarctic krill, *Euphausia superba* Dana (Euphausiacea). J Crustacean Biol. 1991; 11: 236–249. doi: 10.2307/1548361

[37] Cuzin-Roudy J, Buchholz F. Ovarian development and spawning in relation to the moult cycle in Northern krill, *Meganyctiphanes norvegica* (Crustacea: Euphausiacea), along a climatic gradient. Mar Biol. 1999; 133: 267–281. doi: 10.1007/s002270050466

[38] Dalpadado P, Ellertsen B, Johannessen S. Inter-specific variations in distribution, abundance and reproduction strategies of krill and amphipods in the Marginal Ice Zone of the Barents Sea. Deep-Sea Res II. 2008; 55: 2257–2265. doi: 10.1016/j.dsr2.2008.05.015

[39] Mauchline J. The Biology of Mysids and Euphausiids. Blaxter JHS, Russell SFS and Yonge SM, editors. London and New York: Academic Press; 1980.

[40] Krag LA, Herrmann B, Iversen SA, Engås A, Nordrum S, Krafft BA. Size selection of Antarctic krill (*Euphausia superba*) in trawls. PloS one. 2014; 9: e102168 doi: 10.1371/journal.pone.0102168 25105960 10.1371/journal.pone.0102168 PMC4126659

[41] Zuur AF, Ieno EN, Walker NJ, Saveliev AA, Smith GM. Mixed effects models and extensions in ecology with R Gail M, Krickeberg K, Samet JM, Tsiatis A and Wrong W, editors USA: Springer; 2009.

[42] Benaglia T, Chauveau D, Hunter D, Young D. mixtools: An r package for analyzing finite mixture models. J Stat Softw. 2009; 32: 1–29. doi: 10.18637/jss.v032.i06

[43] Gayanilo FC, Sparre P, Pauly D. FAO-ICLARM Stock Assessment Tools II (Fisat II). Revised version. User’s guide. Rome: Food Agriculture Organization of United Nations; 2005.

[44] Bhattacharya C. A simple method of resolution of a distribution into Gaussian components. Biometrics. 1967; 23: 115–135. doi: 10.2307/2528285 6050463

[45] Dalpadado P, Skjoldal HR. Abundance, maturity and growth of the krill species *Thysanoessa inermis* and *T*. *longicaudata* the Barents Sea. Mar Ecol Prog Ser. 1996; 144: 175–183. doi: 10.3354/meps144175

[46] Nelson GA. Fishmethods: Fishery Science Methods and Models in R. 2014 Available: http://CRAN.R-project.org/package = fishmethods.

[47] Anonymous. Environmental conditions in Icelandic waters 2013. Hafrannsóknir. 2014; 175: 5–19.

[48] Reynisson P, Gislason A. Acoustic measurements of euphausiids around Iceland 2011–2014. Hafrannsóknir. 2015; 181: 26–35.

[49] Dalpadado P. Distribution and reproduction strategies of krill (Euphausiacea) on the Norwegian shelf. Polar Biol. 2006; 29: 849–859. doi: 10.1007/s00300-006-0123-8

[50] Dalpadado P, Skjoldal HR. Distribution and life history of krill from the Barents Sea. Polar Res. 1991; 10: 443–460. doi: 10.1111/j.1751-8369.1991.tb00665.x

[51] Falk-Petersen S, Hagen W, Kattner G, Clarke A, Sargent J. Lipids, trophic relationships, and biodiversity in Arctic and Antarctic krill. Can J Fish Aquat Sci. 2000; 57: 178–191. doi: 10.1139/f00-194

[52] Petursdottir H, Falk-Petersen S, Gislason A. Trophic interactions of meso-and macrozooplankton and fish in the Iceland Sea as evaluated by fatty acid and stable isotope analysis. ICES J Mar Sci. 2012; 69: 1277–1288. doi: 10.1093/icesjms/fss125

[53] Agersted MD, Bode A, Nielsen TG. Trophic position of coexisting krill species: a stable isotope approach. Mar Ecol Prog Ser. 2014; 516: 139–151. doi: 10.3354/meps11055

[54] Schmidt K. Food and Feeding in Northern Krill (*Meganyctiphanes norvegica* Sars). Adv Mar Biol. 2010; 57: 127–171. doi: 10.1016/B978-0-12-381308-4.00005-4 20955891 10.1016/B978-0-12-381308-4.00005-4

[55] Pinchuk AI, Hopcroft RR. Egg production and early development of *Thysanoessa inermis* and *Euphausia pacifica* (Crustacea: Euphausiacea) in the northern Gulf of Alaska. J Exp Mar Biol Ecol. 2006; 332: 206–215. doi: 10.1016/j.jembe.2005.11.019

[56] Tarling GA. Population Dynamics of Northern Krill (*Meganyctiphanes norvegica* Sars). Adv Mar Biol. 2010; 57: 59–90. doi: 10.1016/B978-0-12-381308-4.00003-0 20955889 10.1016/B978-0-12-381308-4.00003-0

[57] Falk-Petersen S, Hopkins C. Ecological investigations on the zooplankton community of Balsfjorden, northern Norway: population dynamics of the euphausiids *Thysanoessa inermis* (Krøyer), *Thysanoessa raschii* (M. Sars) and *Meganyctiphanes norvegica* (M. Sars) in 1976 and 1977. J Plankton Res. 1981; 3: 177–192. doi: 10.1093/plankt/3.2.177

[58] Dalpadado P, Ikeda T. Some observations on moulting, growth and maturation of krill (*Thysanoessa inermis*) from the Barents Sea. J Plankton Res. 1989; 11: 133–139. doi: 10.1093/plankt/11.1.133

